# Supplementary material for: Splice-Junction-Based Mapping of Alternative Isoforms in the Human Proteome
Source: Cell Rep. Author manuscript; Available in PMC 2020 Jan 15. (PMC6961840; doi:10.1016/j.celrep.2019.11.026)
Supplement: 2 [file NIHMS1546469-supplement-2.zip › DF1/PXD006675/VentricularSeptum/VentricularSeptum_39_MAPKAPK5_QVIEEQTTSHESTGGAK.pdf]

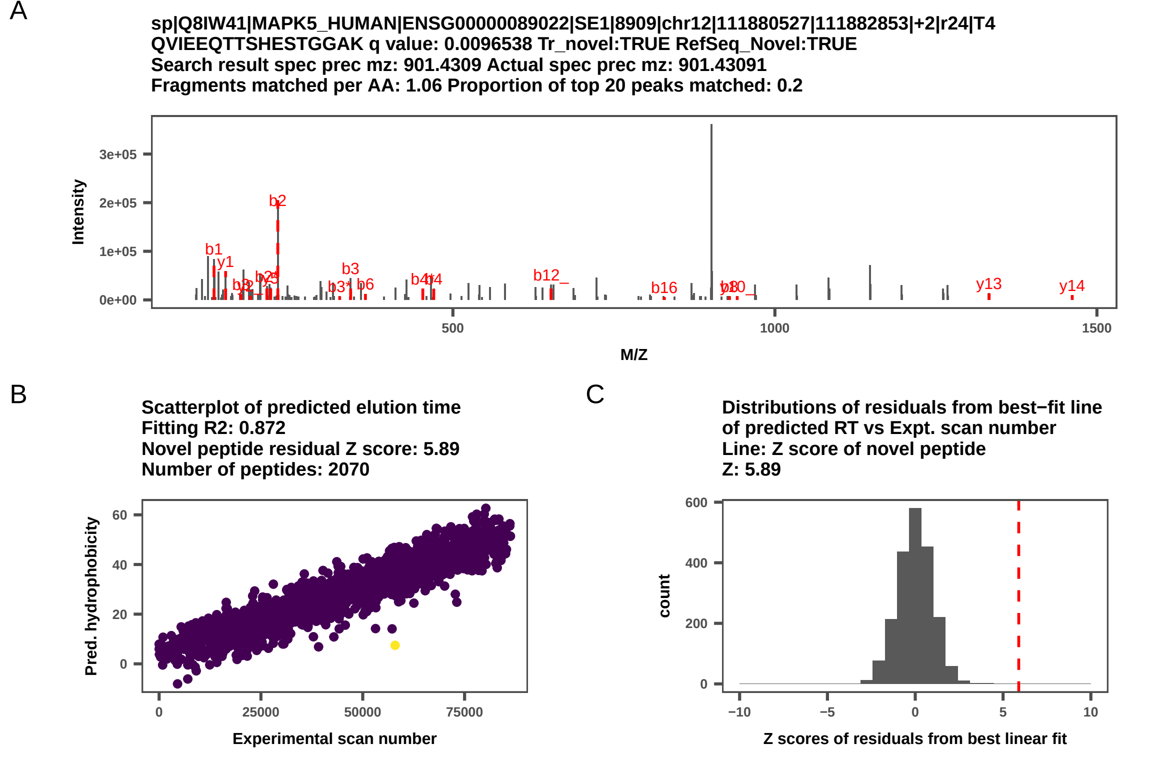

A
sp|Q8IW41|MAPK5_HUMAN|ENSG00000089022|SE1|8909|chr12|111880527|111882853|+2|r24|T4
QVIEEQTTSHESTGGAK q value: 0.0096538 Tr_novel:TRUE RefSeq_Novel:TRUE
Search result spec prec mz: 901.4309 Actual spec prec mz: 901.43091
Fragments matched per AA: 1.06 Proportion of top 20 peaks matched: 0.2
Intensity
3e+05
2e+05
1e+05
0e+00
b2
b1
y1
b3
b6
b12_
b16
y13
y14
500
1000
1500
M/Z
B
Scatterplot of predicted elution time
Fitting R2: 0.872
Novel peptide residual Z score: 5.89
Number of peptides: 2070
Pred. hydrophobicity
60
40
20
0
25000
50000
75000
Experimental scan number
C
Distributions of residuals from best−fit line
of predicted RT vs Expt. scan number
Line: Z score of novel peptide
Z: 5.89
count
600
400
200
−10
−5
5
10
Z scores of residuals from best linear fit
